# The 340B Drug Pricing Program and Management of Advanced Prostate Cancer

**DOI:** 10.1002/cam4.70552

**Published:** 2024-12-30

**Authors:** Kassem S. Faraj, Samuel R. Kaufman, Mary Oerline, Christopher Dall, Arnav Srivastava, Megan E. V. Caram, Vahakn B. Shahinian, Brent K. Hollenbeck

**Affiliations:** ^1^ Dow Division of Health Services Research, Department of Urology University of Michigan Ann Arbor Michigan USA; ^2^ Department of Urology Massachusetts General Hospital Boston Massachusetts USA; ^3^ VA Health Services Research and Development, Center for Clinical Management Research VA Ann Arbor Healthcare System Ann Arbor Michigan USA; ^4^ Division of Hematology/Oncology, Department of Internal Medicine University of Michigan Ann Arbor Michigan USA; ^5^ Division of Nephrology, Department of Internal Medicine University of Michigan Ann Arbor Michigan USA

**Keywords:** androgen receptor antagonists, health policy, healthcare disparities, prostatic neoplasms, social vulnerability

## Abstract

**Introduction:**

Oral targeted therapies are a standard of care for men with advanced prostate cancer. However, these therapies are expensive, which may be a barrier to some, particularly the most economically disadvantaged. Through investment in programs to assist this population, savings generated from the 340B program have the potential to mitigate barriers to initiating treatment with targeted therapies in these men.

**Methods:**

We performed a retrospective study using a 20% national sample of fee‐for‐service Medicare beneficiaries diagnosed with advanced prostate cancer between 2012 and 2019. The outcome was the patient‐level use of a targeted therapy for the first time. This study had two exposures. The first was 340B penetration, representing the percentage of all outpatient hospital revenue in a hospital referral region generated by a 340B hospital. The second was the degree of socioeconomic disadvantage, as measured by the social vulnerability index (SVI). Two separate Cox models were fit to measure relationships between each exposure and use of a targeted therapy. A third model was fitted to assess whether differences in utilization by SVI were mitigated by increasing 340B penetration.

**Results:**

The use of a targeted therapy did not vary with 340B penetration (adjusted HR 1.1, 95% CI 0.96–1.2) for high versus low penetration. Conversely, socioeconomically disadvantaged men were less likely to initiate treatment. Those residing in the third SVI tertile (i.e., most vulnerable) were less likely to start on a targeted therapy compared to men in the first tertile (adjusted HR 0.85, 95% CI 0.78–0.92). However, increasing 340B penetration did not attenuate these differences (Wald test for the interaction term *p* = 0.10).

**Conclusions:**

There was no association between a region's 340B penetration and use of a targeted therapy. Furthermore, although the use of a targeted therapy decreased with increased SVI, the 340B penetration of a region did not reduce this gap.

## 1 Introduction

The management of advanced prostate cancer has evolved from cytotoxic chemotherapy to oral “targeted” therapies [1]. These oral therapies directly act on the androgen pathway and have similar benefits in terms of cancer control as cytotoxic chemotherapy. Importantly, targeted therapies are generally well tolerated and life‐threatening toxicity is uncommon [2, 3, 4], supporting their role as first‐line treatment for both castrate‐sensitive and castrate‐resistant states of the disease [5]. However, these drugs are expensive and associated with significant financial burden [6, 7], which may prohibit access to some men, particularly those with limited financial means. Existing healthcare policy may facilitate access to this important class of medications, thereby eliminating differences in utilization across the spectrum of socioeconomic disadvantage.

The 340B Drug Pricing Program allows participating hospitals to purchase select outpatient medications from the manufacturer at a 30%–70% discount [8]. The program intends that the savings be allocated to better serve disadvantaged patient populations and their communities [9]. Fidelity between the intent of 340B and real‐world application of the savings it generates has been mixed [10, 11]. On one hand, the substantial margin generated from oral specialty drugs for cancer can be deployed to benefit patients in need through expansion of drug assistance programs, care management coordinators, and social work services, among others [12, 13]. On the other hand, downward pressures on hospital finances for clinical care [14, 15, 16] may limit investment of this margin on resources to help disadvantaged populations [17, 18].

To better understand these tradeoffs, we performed a retrospective cohort study assessing relationships between market‐level measures of 340B penetration and socioeconomic disadvantage, and use of targeted therapies in men with advanced prostate cancer. We hypothesized that the use of targeted therapies would increase with higher 340B penetration but decrease with greater socioeconomic disadvantage. Finally, we assessed the relationship of the interaction between the market‐level measures and use of targeted therapies, hypothesizing that increasing 340B penetration would reduce gaps in utilization across the spectrum of socioeconomic disadvantage.

## 2 Methods

Using a 20% national sample of beneficiaries with Traditional Medicare, we performed a retrospective cohort study of men aged 66 years or older with advanced prostate cancer between January 1, 2012 and December 31, 2019. Men with advanced prostate cancer (*n* = 43,558) were identified based on their initiation of chronic androgen deprivation therapy, defined by bilateral orchiectomy or at least 6 months of continuous treatment with a gonadotropin‐releasing hormone analog in the absence of local treatment (i.e., surgery or radiation in the 12‐month period before or after initiation of androgen deprivation) [19, 20]. This likely included men with metastatic castrate‐resistant, metastatic castrate‐sensitive, nonmetastatic castrate‐resistant disease, biochemically recurrent prostate cancer, or nonmetastatic castrate‐sensitive disease. Those managed with a targeted therapy (i.e., abiraterone, enzalutamide, apalutamide, and darolutamide) were identified using Medicare Part D. We required men to have continuous enrollment in Medicare Parts A, B, D for at least 12 months after the start of androgen deprivation therapy. The primary outcome was the use of any oral targeted therapy for the first time, measured at the patient‐level.

**FIGURE 1:**
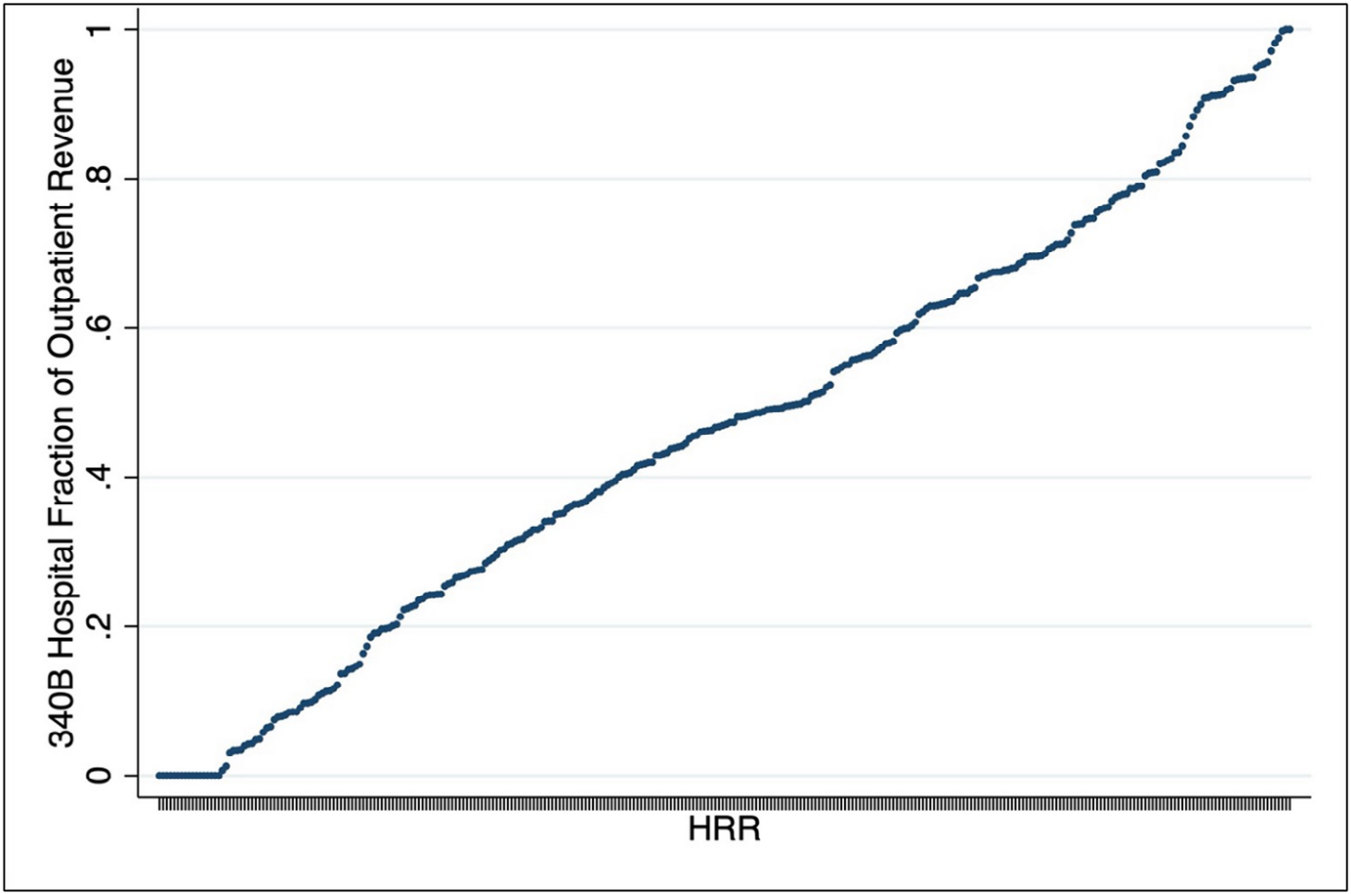
Outpatient 340B penetration by hospital referral region.

This study had two exposures. The first was the 340B penetration, measured at the level of the hospital referral region (HRR). Annual participation in the 340B program was established for every hospital in the United States using Health Resources and Services Administration data [21]. In each HRR, the percentage of the total outpatient revenue occurring at 340B hospitals was defined using the Hospital Provider Cost Report [22]. As illustrated in Figure 1, the percentage of outpatient revenue at 340B hospitals at the HRR‐year level ranged from 0% to 100%. While not a direct measure of receiving the medication from the hospital, we would expect men living in HRRs with low 340B penetration to be less likely to get drugs through the program than those residing in regions with high 340B penetration. HRRs were sorted into tertiles—low (≤ 37%), medium (> 37% and ≤ 57%), and high (> 57% and ≤ 100%). The second exposure was socioeconomic disadvantage, described by the social vulnerability index (SVI), and was measured at the zip code level. This measure, developed by the U.S. Centers for Disease Control and Prevention, characterizes markets by several social constructs [23] and is measured on a scale from 0 to 1, with 1 representing the most socially vulnerable areas. The SVI was then sorted into tertiles—low (≤ 0.39), medium (> 0.39 and ≤ 0.60), and high (> 0.60 and ≤ 1).

### 2.1 Statistical Analysis

Patient characteristics across 340B penetration and SVI were compared using Pearson's Chi‐squared test and analysis of variance (ANOVA) test for discrete and continuous data, respectively. The primary outcome—the day of a prescription fill for a targeted therapy for the first time—was assessed using a cumulative hazard function, with time zero denoted as the day started on androgen deprivation therapy. Men were followed up to 4 years and were censored at the end of 4 years or death. We assessed the association between 340B penetration tertiles and use of a targeted therapy using a Cox model, with clustering at the HRR. This model was adjusted for patient age, comorbidity [24], race, rural residence, time on androgen‐deprivation therapy, year of androgen deprivation therapy initiation, dual‐eligibility, and market characteristics (e.g., supply of hospital beds, Medicare managed care penetration). A similar modeling framework was used to assess the relationship between SVI tertiles and the use of a targeted therapy. To test whether the association between SVI and treatment varied with 340B penetration, a separate model was fitted to assess the relationship of the interaction between SVI and 340B penetration and use of a targeted therapy.

All analyses were carried out using Stata 17 (College Station, TX). Tests were two‐sided with probability of type 1 error (*α*) set at 0.05. The study protocol was judged to be exempt by the University of Michigan institutional review board and the requirement for informed consent was waived.

## 3 Results

**FIGURE 2:**
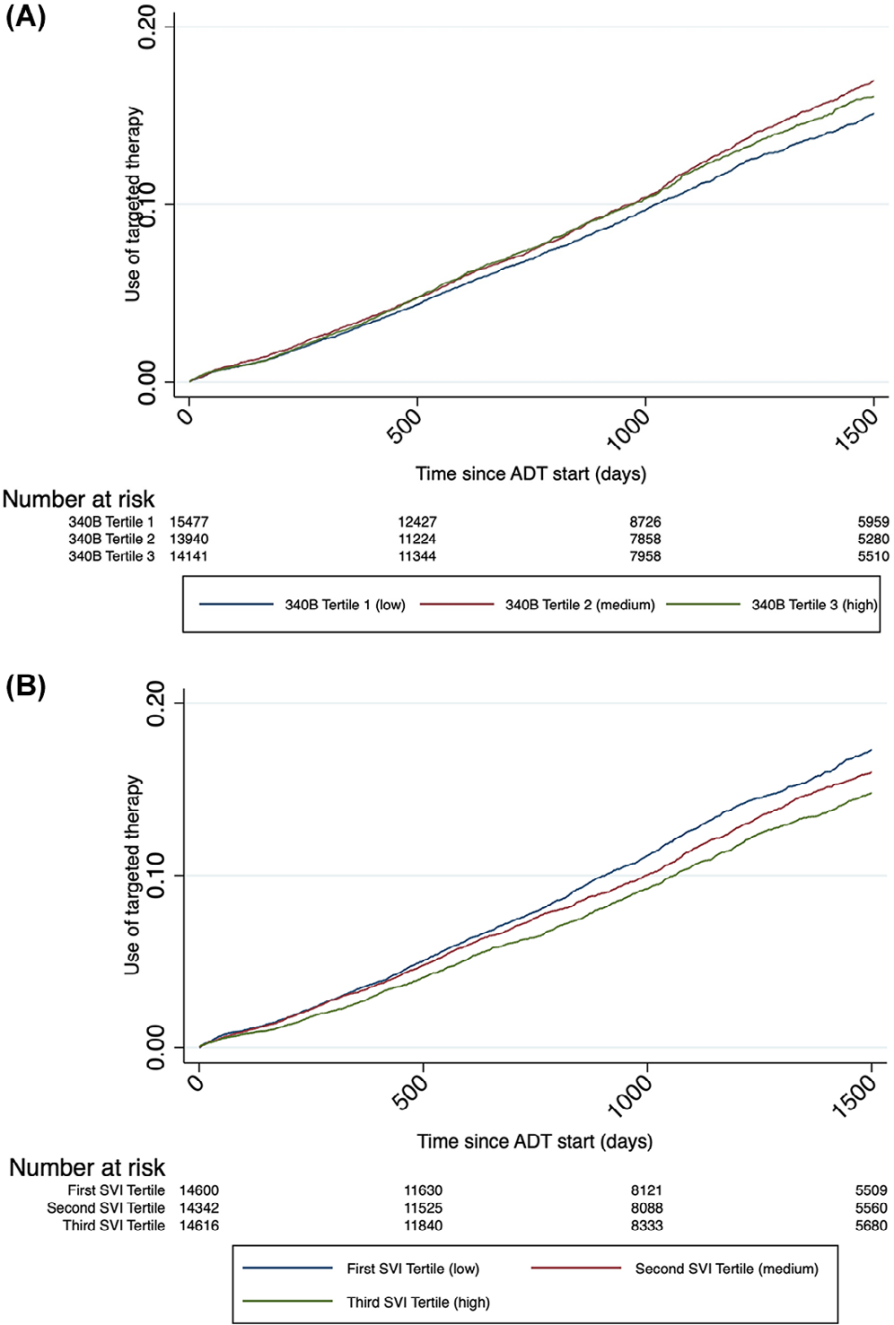
Use of therapy since start of ADT in advanced prostate cancer cohort. (A) Across 340B penetration tertiles. (B) Across social vulnerability tertiles.

Baseline characteristics by 340B penetration are shown in Table 1. Notably, men in the third tertile (i.e., highest 340B penetration) more often resided in a rural location and more often had concurrent coverage for Medicaid. As shown in Table 2, there were also modest differences across SVI tertiles. For example, those in the third tertile (i.e., most vulnerable) were more often Black and dual‐eligible, while men in the first tertile (i.e., least vulnerable) were less likely to reside in rural areas. Crude cumulative incidence plots demonstrated that targeted therapy use was lowest in markets least penetrated by 340B (Figure 2A, log rank *p* = 0.007). Additionally, men residing in the most disadvantaged areas as measured by the SVI were the least likely to initiate a targeted therapy (Figure 2B, log rank, *p* < 0.001).

**TABLE 1:** Characteristics by 340B penetration of hospital referral regions.

Number of advanced prostate cancer patients	First tertile	Middle tertile	Third tertile	*p*
	15,477	13,940	14,141	
Age, mean (SD)	80 (7)	79 (7)	80 (7)	0.02
Fraction of 340B outpatient revenue, mean (SD)	0.17 (0.09)	0.47 (0.06)	0.76 (0.14)	< 0.001
Race				
White	13,392 (86)	11,719 (84)	11,680 (83)	< 0.001
Black	1521 (10)	1785 (13)	1684 (12)	
Other	564 (4)	436 (3)	777 (5)	
Comorbidity score (%)				
0	11,093 (72)	9964 (72)	10,069 (71)	0.95
1	1560 (10)	1431 (10)	1430 (10)	
2+	2824 (18)	2545 (18)	2642 (19)	
Hospital bed per 100,000				
Low (≤ 3340)	5401 (35)	5309 (38)	3904 (28)	< 0.001
Intermediate	4894 (32)	4101 (29)	5376 (38)	
High (≥ 6342)	5182 (33)	4530 (33)	4861 (34)	
Medicare advantage penetration				
Low (≤ 13.5%)	5731 (37)	4426 (32)	4462 (32)	< 0.001
Intermediate	5230 (34)	4894 (35)	4234 (30)	
High (≥ 25.0%)	4516 (29)	4620 (33)	5445 (38)	
Rural residence (%)	3413 (22)	3399 (24)	3702 (26)	< 0.001
Dual‐eligible patients (%)	1099 (7)	1064 (8)	1630 (12)	< 0.001

**TABLE 2:** Characteristics by social vulnerability index.

Number of advanced prostate cancer patients	First tertile	Middle tertile	Third tertile	*p*
	14,600	14,342	14,616	
Age, mean (SD)	80 (7)	80 (7)	79 (7)	0.001
Race				
White	13,607 (93)	12,731 (89)	10,453 (71)	< 0.001
Black	623 (4)	1160 (8)	3208 (22)	
Other	370 (3)	451 (3)	955 (7)	
Comorbidity score (%)				
0	10,666 (73)	10,258 (72)	10,202 (70)	< 0.001
1	1384 (9)	1495 (10)	1542 (10)	
2+	2550 (18)	2589 (18)	2872 (20)	
Hospital bed per 100,000				
Low (≤ 3340)	4900 (33)	5356 (37)	4358 (30)	< 0.001
Intermediate	5349 (37)	4463 (31)	4559 (31)	
High (≥ 6342)	4351 (30)	4523 (32)	5699 (39)	
Medicare advantage penetration				
Low (≤ 13.5%)	4387 (30)	4763 (33)	5469 (37)	< 0.001
Intermediate	4860 (33)	4891 (34)	4607 (32)	
High (≥ 25.0%)	5353 (37)	4688 (33)	4540 (31)	
Rural residence (%)	1878 (13)	4041 (28)	4595 (31)	< 0.001
Dual‐eligible patients (%)	558 (4)	1025 (7)	2210 (15)	< 0.001

After adjusting for differences in patients and markets (Table 3), 340B penetration was not associated with use of a targeted therapy. Compared to men residing in areas with the lowest 340B penetration, those in the highest 340B penetration had similar utilization (adjusted HR 1.1, 95% CI 0.96–1.2). Conversely, SVI was inversely associated with the use of a targeted therapy. Men living in the most disadvantaged areas (i.e., the third tertile) were less likely to start on a targeted therapy compared to those in the first tertile (HR 0.85, 95% CI 0.78–0.92). In a separate model, the interaction between SVI and 340B penetration on the use of therapy was tested. This interaction was not statistically significant (Wald Test *p* = 0.10). This suggests that 340B penetration minimally affects the association between SVI and the use of a targeted therapy.

**TABLE 3:** Cox models assessing the use of a targeted therapy adjusted for patient and market characteristics.^a^

	Hazard ratio (95% CI)	*p*
Model 1—340B penetration		
Use of targeted therapy		
340B penetration tertile 1 (low)	Referent	
340B penetration tertile 2	1.1 (0.99–1.2)	0.070
340B penetration tertile 3 (high)	1.1 (0.96–1.2)	0.27
Model 2—SVI		
Use of targeted therapy		
SVI tertile 1 (least disadvantaged)	Referent	
SVI tertile 2	0.93 (0.87–1.1)	0.10
SVI tertile 3 (most disadvantaged)	0.85 (0.78–0.92)	< 0.001

^a^ Models were adjusted for patient age, comorbidity, race, rural residence, time on androgen‐deprivation therapy, year of androgen deprivation therapy initiation, dual‐eligibility and market characteristics (i.e., supply of hospital beds, Medicare managed care penetration).

## 4 Discussion

Men with advanced prostate cancer living in HRRs least penetrated by the 340B program were just as likely to start a targeted therapy as those residing in the highest penetrated areas. Men with advanced prostate cancer living in the most socioeconomically disadvantaged areas were less likely to start treatment with a targeted therapy. The gap in utilization between levels of disadvantage, as measured by the SVI, was not significantly mitigated by greater 340B penetration.

The 340B program has been criticized for lack of oversight [25] and how participating hospitals have used savings garnered from the program [11, 17, 26]. For instance, while the program was intended for hospitals to stretch federal resources to provide for communities [21, 27, 28], empirical findings have been mixed. On the positive side, prior work has demonstrated the 340B program has been associated with net benefits for communities, particularly for disadvantaged patients. For instance, hospital participation in the program has been associated with providing more charity care and low‐profit inpatient services [29], prescription support services, free and discounted medications, patient assistance programs, free immunizations, among others [12]. However, on the negative side, where some claim that the program has not been associated with net benefits for communities and patients, studies have demonstrated that participation in the program did not lead to increased safety‐net provisions [10] or improved health outcomes in low‐income patients [10]. While one study showed that 340B participation was associated with increased charity care spending, this was offset by reductions in other community benefit programs [11]. Furthermore, others have shown that the 340B program has facilitated financially driven decisions for hospitals, as participating hospitals are less likely to use less expensive biosimilar medications [18], greater volumes of therapies [10, 30], and preferentially expand contract pharmacy and affiliated clinic reach to affluent areas [17, 26]. In this study, we assessed 340B penetration at the market‐level, by comparing HRRs based on 340B outpatient revenue. We found that the use of therapy did not vary by the 340B penetration of a region. Furthermore, while the use of therapy decreased with a patient's SVI, greater 340B penetration in a region was not associated with a reduction of this gap.

This study should be interpreted within the context of limitations. First, we did not directly assess the effect of the 340B program itself. Nonetheless, our market‐level exposure (340B outpatient revenue in a HRR/total outpatient revenue in a HRR) represented a substantial gradient across the three tertiles of 340B penetration, supporting plausible differences in probabilities of men in these regions interacting with and benefitting from the program. Further, the 340B program intended to stretch resources to improve the care of the broader communities that these hospitals serve [27, 28, 31], providing a conceptual basis for a market‐level analysis. Second, a strength of this study is that it is population‐based using national Medicare. However, because this study relies on claims, we were unable to assess differences in burden of metastases and/or disease trajectory across tertiles of 340B penetration. However, targeted therapies for advanced prostate cancer were approved by the Food and Drug Administration solely for metastatic castrate‐resistant disease until 2018 and thus this group of men (i.e., 4163 of 5855 men initiating therapy) likely constitutes the majority of the men in this study. Further, while differences in disease burden vary from patient to patient, we would not expect meaningful differences across large populations of men across HRRs. Next, as the SVI was derived at the zip code level, it is possible that some men's degree of vulnerability may be misclassified based on their place of residence. Nonetheless, prior work has demonstrated that such measures of socioeconomic status are associated with patient outcomes [32, 33], supporting its construct validity, albeit imperfectly. We also did not evaluate whether patients received primary chemotherapy after initiating androgen deprivation therapy, but we would not expect this to vary by groups according to 340B penetration. Finally, with the recent FDA approval of enzalutamide for biochemically recurrent prostate cancer in 2023, these men may also be affected by the program, but may not be included in our algorithm, since we required a history of chronic androgen deprivation therapy. However, this study included men diagnosed with advanced disease up to December 2019. Thus, these men would not have been eligible to receive a targeted therapy during this timeframe.

This study has implications for men with advanced prostate cancer and policy reform discussions around the 340B program. Our findings that SVI was inversely related to the use of a targeted therapy confirms prior work demonstrating that access to a standard of care varies by socioeconomic status, both in [13, 34] and outside of [35, 36] the prostate cancer context. While the mechanism by which this outcome varied was unclear (e.g., medication costs, physician beliefs, patient preference), our findings suggest an opportunity for the 340B program to further close the observed gaps in these communities. As high cost‐sharing for targeted therapies in men with advanced prostate cancer can be a barrier to access [6, 7], participating 340B hospitals could implement programs to assist vulnerable members of their community. Implementing screening programs to detect patients at risk for access/financial toxicity may allow high‐yield referrals to manufacturers for discounts or identify those who might benefit most from free and/or discounted prescriptions, as some have done [12]. Investing resources from 340B in a manner linked most tightly with need has the potential to close gaps in utilization motivated by financial and/or social barriers to access. Furthermore, men who are socioeconomically disadvantaged are often diagnosed with advanced disease and generally experience worse outcomes compared to the general population [37, 38], necessitating the need for policy reform to mitigate these disparities.

## 5 Conclusions

Market‐level 340B penetration, as measured by the proportion of outpatient revenue in a HRR by a participating hospital, was not associated with use of a targeted therapy in men with advanced prostate cancer. Conversely, increasing social vulnerability, as measured by the SVI, was associated with lower use of these therapies. Increasing market‐level 340B did not mitigate the gaps in utilization across levels of social vulnerability. Future work should explore mechanisms underlying differences in utilization of targeted therapies among socially vulnerable men with advanced prostate cancer.

## Author Contributions

**Kassem S. Faraj:** conceptualization (equal), investigation (equal), supervision (equal), validation (equal), writing – original draft (equal), writing – review and editing (equal). **Samuel R. Kaufman:** methodology (equal), resources (equal), software (equal), supervision (equal), writing – review and editing (equal). **Mary Oerline:** methodology (equal), resources (equal), software (equal), validation (equal), writing – review and editing (equal). **Christopher Dall:** investigation (equal), methodology (equal), writing – review and editing (equal). **Arnav Srivastava:** investigation (equal), resources (equal), writing – review and editing (equal). **Megan E. V. Caram:** conceptualization (equal), supervision (equal), validation (equal), writing – review and editing (equal). **Vahakn B. Shahinian:** conceptualization (equal), funding acquisition (equal), investigation (equal), validation (equal), writing – review and editing (equal). **Brent K. Hollenbeck:** funding acquisition (equal), investigation (equal), methodology (equal), project administration (equal), resources (equal), supervision (equal), writing – original draft (equal), writing – review and editing (equal).

## Conflicts of Interest

The authors declare no conflicts of interest.

## Precis

Men with advanced prostate cancer who received care in markets highly penetrated by the 340B program had similar utilization of oral specialty drugs as those cared for in markets with low 340B penetration. While socially vulnerable areas were associated with significantly lower use of oral specialty drugs, 340B penetration did not attenuate these differences. Future work should explore mechanisms underlying differences in utilization of targeted therapies among socially vulnerable men with advanced prostate cancer.

## Data Availability

This study used national Medicare claims data provided by the Centers for Medicare & Medicaid Services (CMS) under license or by permission.
